# Case Report: Neonatal Massive Pneumothorax Resulting in Compression Atelectasis Treated by Ultrasound-Guided Pleural Puncture Therapy: A Typical Case Based on Lung Ultrasound Finding

**DOI:** 10.3389/fped.2021.779615

**Published:** 2021-11-30

**Authors:** Jing Liu, Ru-Xin Qiu, Ying Liu

**Affiliations:** Department of Neonatology and NICU, Beijing Chaoyang District Maternal and Child Healthcare Hospital, Beijing, China

**Keywords:** atelectasis, pneumothorax, neonate, lung ultrasound, imaging, case report

## Abstract

Atelectasis is a complication of different pulmonary diseases; however, neonatal compression atelectasis due to pneumothorax is rarely reported in the literature. Recently, we encountered a typical case of atelectasis. A preterm infant was admitted to the neonatal intensive care unit owing to severe respiratory distress. Lung ultrasound examination confirmed severe pneumothorax and large area of atelectasis. Lung re-expansion occurred when the air was drained from the pleural cavity.

## Introduction

Atelectasis is a common complication of neonatal lung diseases and is one of the causes of dyspnea, weaning difficulty from mechanical ventilation, and bronchopulmonary dysplasia (BPD) in premature infants (1). A common type of neonatal atelectasis is obstructive atelectasis. Although a large pleural effusion or pneumothorax (PTX) can theoretically cause compression atelectasis, it is rarely seen in clinically practice and is rarely reported in the literature. Previously, we reported a case of neonatal compression atelectasis caused by congenital massive pleural effusion and treatment after pleural puncture drainage under ultrasound guidance (2). In order to further deepen the understanding and correct management of neonatal oppressive atelectasis, we report a case of compression atelectasis caused by severe PTX that was resolved after ultrasound-guided pleural puncture therapy.

## Case Presentation

A female newborn infant delivered vaginally at gestational age 34^+1^ weeks with birth weight 1,900 g and mild asphyxia at birth with Apgar scores 6, 9, and 10 at 1, 5, and 10 min, respectively, was hospitalized at our neonatal intensive care unit (NICU) due to severe respiratory distress 20 min after birth. Physical examination showed significant prominence in the right thorax, and respiratory difficulty presented as respiratory frequency of more than 80 breaths/min accompanied by grunting, flaring, and retracting.

**Figure 1:**
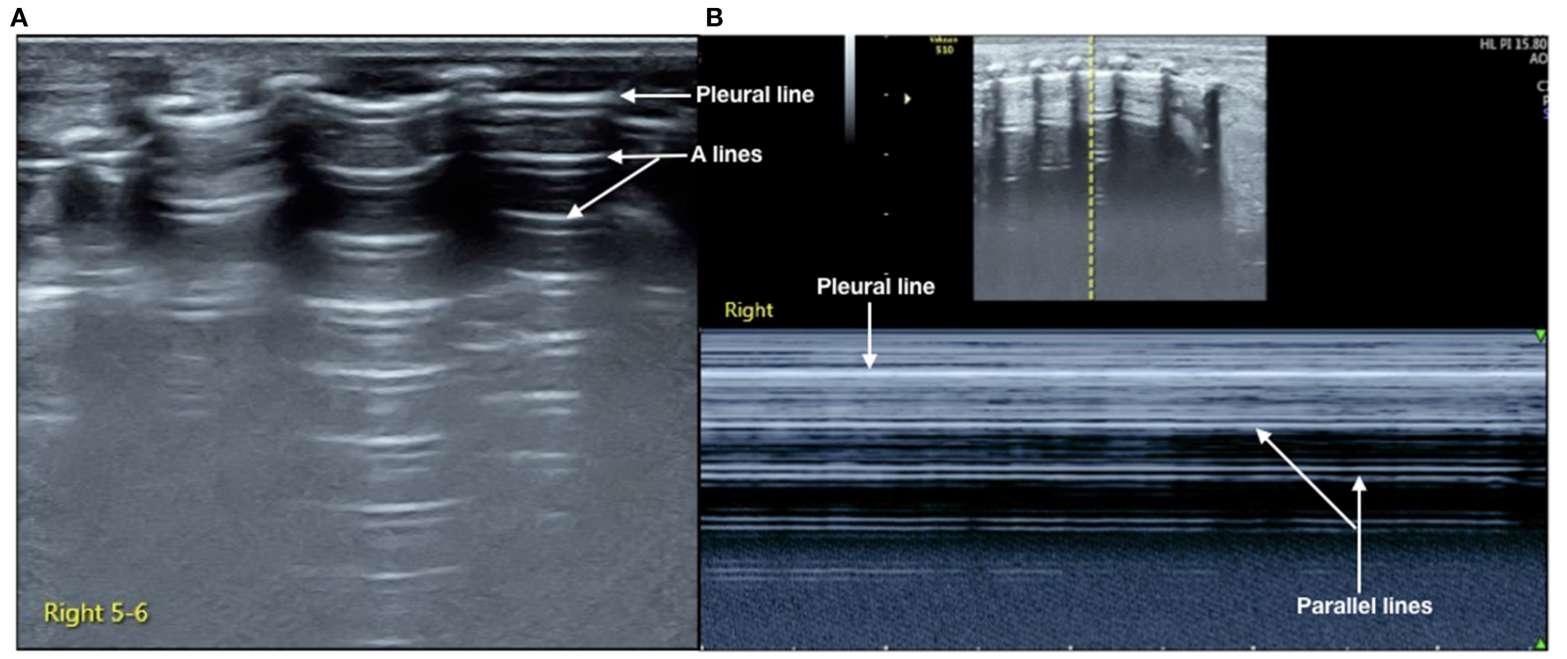
Lung ultrasonography (LUS) of the right chest. LUS examination on anterior, subaxillary, and posterior chest away from the spine. **(A)** B-mode ultrasound, we can see that the pleural line and A-line clearly existed but no B-line was found. **(B)** M-mode ultrasound; it presented as a stratosphere sign. It was confirmed as the severe PTX according to the *International Expert Consensus and Recommendations for Neonatal Pneumothorax Ultrasound Diagnosis* (3).

**Figure 2:**
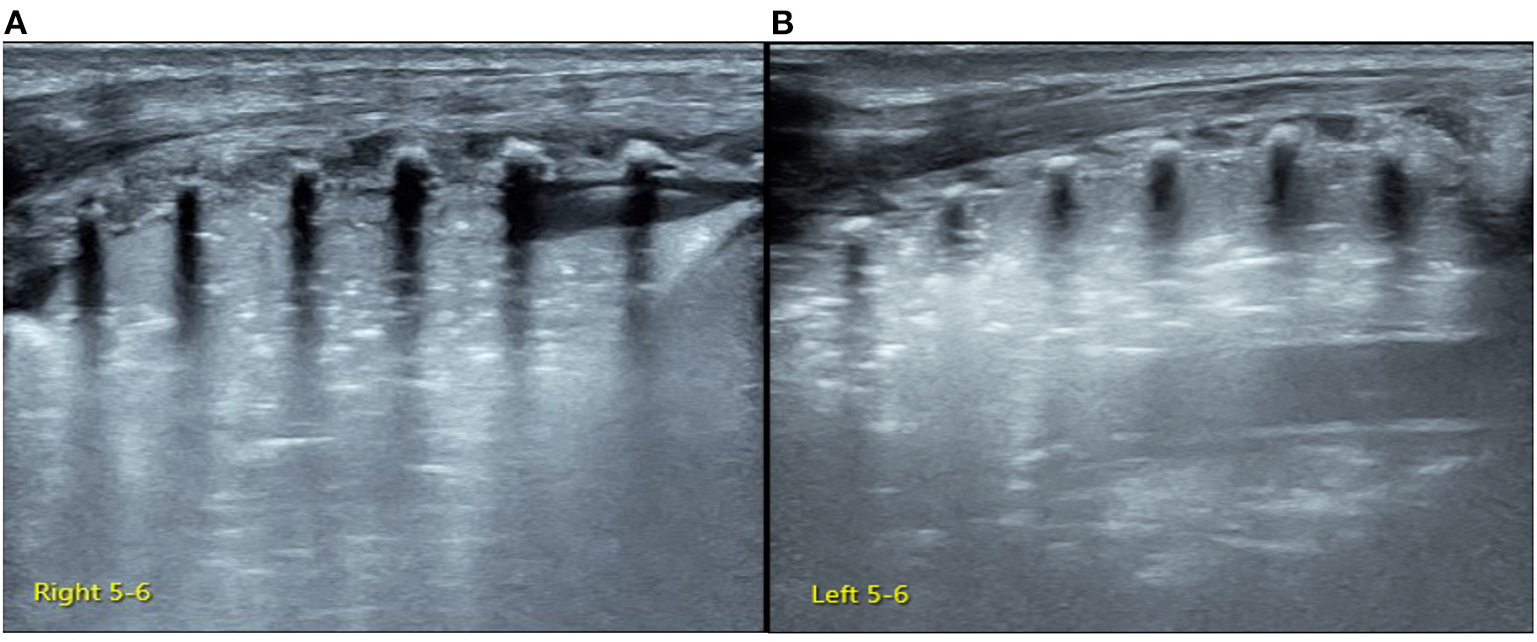
Lung ultrasonography (LUS) of the posterior chest. LUS examination of the right back close to the spine **(A)** and the left back **(B)** presents large consolidation with air bronchograms. Lung pulse is found under real-time ultrasound, which confirms the presence of large area atelectasis in bilateral lungs.

**Figure 3:**
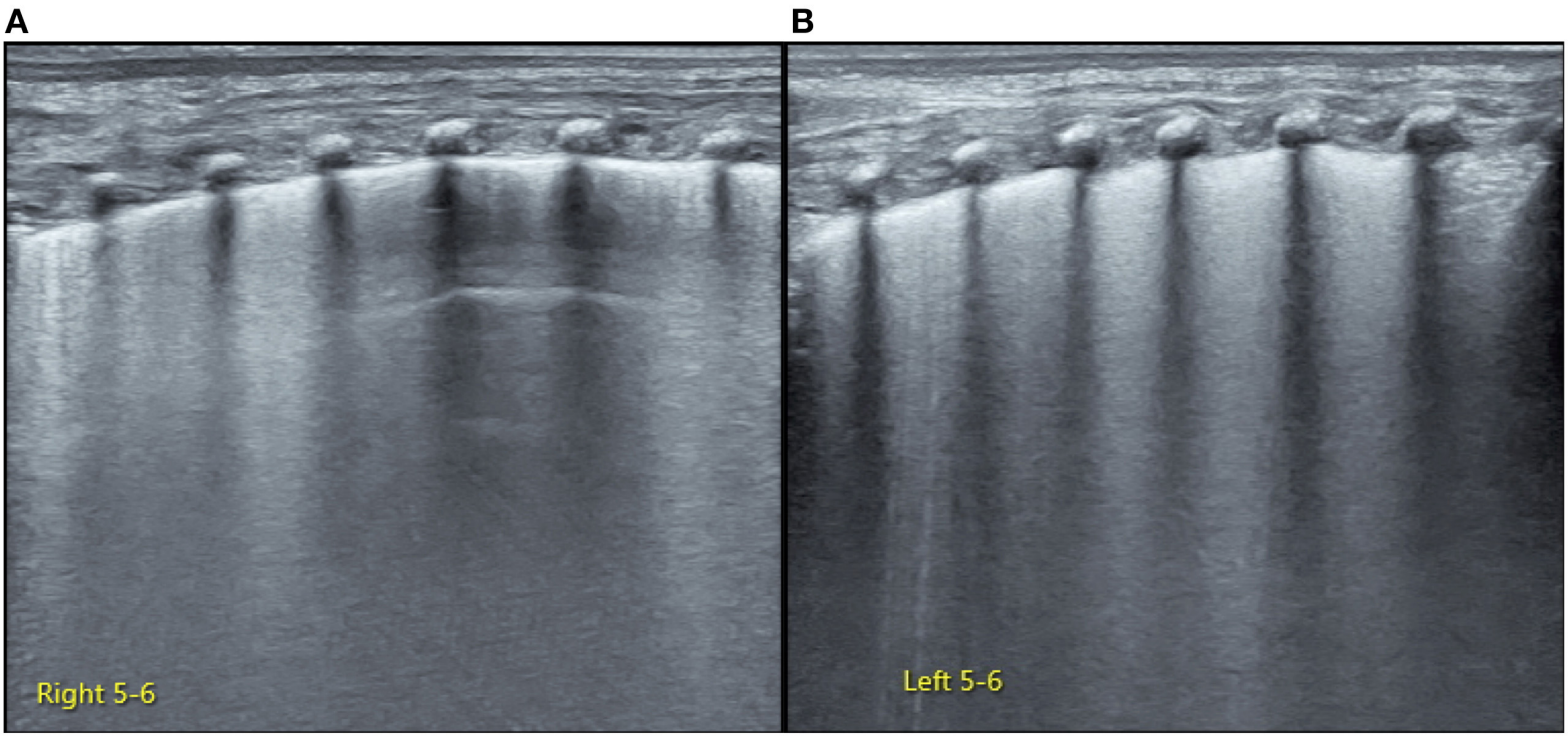
Lung ultrasonography (LUS) examination after thoracocentesis. LUS examination shows resolution of atelectasis in bilateral lungs after pleural puncture therapy. The right upper-lung and lower-lung field mainly present as confluent B-line (but the A-line can be found in the middle-lung field), which suggests some degree of lung edema **(A)**. The left lung in the last intercostal space reveals snowflake signs while the remaining intercostal spaces present as ground-glass opacities, which are characteristic of respiratory distress syndrome (RDS) (6, 7) **(B)**.

Lung ultrasonography (LUS) was performed using GE Voluson S10 with a frequency of an ML 6-15 linear probe (GE Healthcare, USA). On admission, the infant was placed in a lateral position. While examining the right lung, when the probe was placed in the anterior chest, subaxillary region, and posterior chest away from the spine, we found that the pleural line and A-line were clearly displayed under B-mode ultrasound. The stratosphere sign was presented under M-mode ultrasound (Figures 1A,B), while the lung sliding disappeared under real-time ultrasound, which was confirmed as severe PTX in the right chest (3). However, when the probe was placed close to the spine, the LUS revealed large-area lung consolidations with air bronchograms (Figure 2A), which was confirmed as atelectasis (4, 5). LUS of the left lung revealed severe pulmonary atelectasis (Figure 2B). The infant was placed in the left lateral position to facilitate puncture to drain gas from the right chest cavity. Thoracentesis was performed using an angiocatheter connected to a 20-ml syringe one by one in the right fifth to sixth intercostal space along the middle axillary line under LUS guidance (3). After a total of 520 ml of gas was removed, the right PTX disappeared thoroughly, as did bilateral atelectasis, and the LUS manifested as B-lines in his right lung, while it presented as ground glass opacity sign and snowflake signs in his left lung, which was confirmed as mild-moderate respiratory distress syndrome (RDS) (6, 7) (Figure 3).

Other laboratory tests, including blood routine, C-reactive protein, and procalcitonin levels, performed after admission showed no abnormal findings. The patient was discharged 10 days after appropriate treatment (this included 12 h of invasive mechanical ventilation and 24 h of BiCPAP therapy) because of existing RDS. The infant was followed up to 6 months after birth and showed good growth and development without abnormalities of the respiratory and other abnormalities. Written informed consent was obtained from the parents of the infant for publication of any potentially identifiable images or data included in this article.

## Discussion

PTX is a common severe disease in newborn infants, with a reported incidence of 0.56% in the neonatal population (8). In hospitalized neonates, its incidence increased to 4.5% (9), an eight-fold increase. Moreover, PTX causes a 5.27-fold (95% CI = 1.96–14.17) increase in mortality (10), while the incidence of BPD increases 4.28-fold in survivors (11). Therefore, neonatologists have always been highly concerned about the diagnosis and treatment of PTX.

PTX is not an independent disease, but one of the serious complications of other lung diseases.

As in this late-preterm infant, the atelectasis disappeared after pleural puncture therapy, following which the LUS presented as RDS manifestation (5–7). It indicated that the PTX was caused by RDS and was suggestive of a type of compression atelectasis caused by the severe PTX. Owing to the presence of RDS, the patient subsequently received invasive or non-invasive respiratory support and was cured thereafter. This rare case indicates that in critically ill infants, when PTX is diagnosed, further evaluation is required to rule out other complications such as atelectasis.

Using LUS to diagnose neonatal PTX and other pulmonary diseases is more sensitive, accurate, and reliable as compared to using radiography (12, 13). Therefore, in our NICU, LUS has replaced radiography in the past 5 years, not only for the diagnosis of PTX but also for the diagnosis of various common neonatal lung diseases (14, 15), which greatly improves the prognosis of newborn infants.

Traditionally, thoracentesis of PTX is performed in the second intercostal space at the midclavicular line or fourth to fifth intercostal space at the midaxillary line with the needle pointed toward the opposite shoulder with a repeat chest radiograph after the procedure. However, ultrasound-guided pleural puncture is not always subject to such rules with obvious disadvantages (3). Ultrasound-guided thoracentesis can be performed in any site where gas accumulation is seen (3). Systematic reviews revealed no significant differences between needle aspiration and chest tube placement with regard to safety and rates of immediate success; however, needle aspiration is associated with lesser pain and shorter duration of hospital stay than chest tube thoracotomy (16). Hence, we selected angiocatheter puncture, which showed better results in this patient.

## Data Availability Statement

The raw data supporting the conclusions of this article will be made available by the authors, without undue reservation.

## Ethics Statement

Written informed consent was obtained from her parents for the publication of any potentially identifiable images or data included in this article.

## Author Contributions

JL contributed to the study conception, ultrasound examination, data analysis, and wrote and approved the manuscript. R-XQ contributed to clinical data analysis, manuscript preparation, and approval of the final manuscript. YL contributed to clinical data analysis, manuscript preparation, and approval of the final manuscript. All authors contributed to the article and approved the submitted version.

## Funding

This work was supported by the Social Development Projects, Beijing Chaoyang District Bureau of Science, Technology and Information (CYSF1922).

## Conflict of Interest

The authors declare that the research was conducted in the absence of any commercial or financial relationships that could be construed as a potential conflict of interest.

## Publisher's Note

All claims expressed in this article are solely those of the authors and do not necessarily represent those of their affiliated organizations, or those of the publisher, the editors and the reviewers. Any product that may be evaluated in this article, or claim that may be made by its manufacturer, is not guaranteed or endorsed by the publisher.

## Abbreviations

BPD: bronchopulmonary dysplasia
PTX: pneumothorax
NICU: neonatal intensive care unit
LUS: lung ultrasonography
RDS: respiratory distress syndrome.
